# Association of the *CHEK2* c.1100delC variant, radiotherapy, and systemic treatment with contralateral breast cancer risk and breast cancer-specific survival

**DOI:** 10.21203/rs.3.rs-2569372/v1

**Published:** 2023-02-13

**Authors:** Anna Morra, Maartje A. C. Schreurs, Irene L. Andrulis, Hoda Anton-Culver, Annelie Augustinsson, Matthias W. Beckmann, Sabine Behrens, Stig E. Bojesen, Manjeet K. Bolla, Hiltrud Brauch, Annegien Broeks, Saundra S. Buys, Nicola J. Camp, Jose E. Castelao, Melissa H. Cessna, Jenny Chang-Claude, Wendy K. Chung, NBCS Collaborators, Sarah V. Colonna, Fergus J. Couch, Angela Cox, Simon S. Cross, Kamila Czene, Mary B. Daly, Joe Dennis, Peter Devilee, Thilo Dörk, Alison M. Dunning, Miriam Dwek, Douglas F. Easton, Diana M. Eccles, Mikael Eriksson, D. Gareth Evans, Peter A. Fasching, Tanja N. Fehm, Jonine D. Figueroa, Henrik Flyger, Marike Gabrielson, Manuela Gago-Dominguez, Montserrat García-Closas, José A. García-Sáenz, Jeanine Genkinger, Felix Grassmann, Melanie Gündert, Eric Hahnen, Christopher A. Haiman, Ute Hamann, Patricia A. Harrington, Jaana M. Hartikainen, Reiner Hoppe, John L. Hopper, Richard S. Houlston, Anthony Howell, ABCTB Investigators, kConFab Investigators, Anna Jakubowska, Wolfgang Janni, Helena Jernström, Esther M. John, Nichola Johnson, Michael E. Jones, Vessela N. Kristensen, Allison W. Kurian, Diether Lambrechts, Loic Le Marchand, Annika Lindblom, Jan Lubiński, Michael P. Lux, Arto Mannermaa, Dimitrios Mavroudis, Anna Marie Mulligan, Taru A. Muranen, Heli Nevanlinna, Ines Nevelsteen, Patrick Neven, William G. Newman, Nadia Obi, Kenneth Offit, Andrew F. Olshan, Tjoung-Won Park-Simon, Alpa V. Patel, Paolo Peterlongo, Kelly-Anne Phillips, Dijana Plaseska-Karanfilska, Eric C. Polley, Nadege Presneau, Katri Pylkäs, Brigitte Rack, Paolo Radice, Muhammad U. Rashid, Valerie Rhenius, Mark Robson, Atocha Romero, Emmanouil Saloustros, Elinor J. Sawyer, Rita K. Schmutzler, Sabine Schuetze, Christopher Scott, Mitul Shah, Snezhana Smichkoska, Melissa C. Southey, William J. Tapper, Lauren R. Teras, Rob A.E.M. Tollenaar, Katarzyna Tomczyk, Ian Tomlinson, Melissa A. Troester, Celine M. Vachon, Elke M. van Veen, Qin Wang, Camilla Wendt, Hans Wildiers, Robert Winqvist, Argyrios Ziogas, Per Hall, Paul D.P. Pharoah, Muriel A. Adank, Antoinette Hollestelle, Marjanka K. Schmidt, Maartje J. Hooning

**Affiliations:** The Netherlands Cancer Institute; Erasmus MC Cancer Institute; Lunenfeld-Tanenbaum Research Institute of Mount Sinai Hospital; University of California Irvine; Lund University; University Hospital Erlangen; German Cancer Research Center (DKFZ); Copenhagen University Hospital; University of Cambridge; Dr. Margarete Fischer-Bosch-Institute of Clinical Pharmacology; The Netherlands Cancer Institute; University of Utah; University of Utah; Instituto de Investigación Sanitaria Galicia Sur (IISGS), Xerencia de Xestion Integrada de Vigo-SERGAS; Intermountain Healthcare; German Cancer Research Center (DKFZ); Columbia University; University of Utah; Mayo Clinic; University of Sheffield; University of Sheffield; Karolinska Institutet; Fox Chase Cancer Center; University of Cambridge; Leiden University Medical Center; Hannover Medical School; University of Cambridge; University of Westminster; University of Cambridge; University of Southampton; Karolinska Institutet; University of Manchester, Manchester Academic Health Science Centre; University Hospital Erlangen; University Hospital Düsseldorf, Heinrich-Heine University Düsseldorf; The University of Edinburgh; Copenhagen University Hospital; Karolinska Institutet; Fundación Pública Galega de Medicina Xenómica, Instituto de Investigación Sanitaria de Santiago de Compostela (IDIS), Complejo Hospitalario Universitario de Santiago, SERGAS; National Cancer Institute, National Institutes of Health, Department of Health and Human Services; Instituto de Investigación Sanitaria San Carlos (IdISSC), Centro Investigación Biomédica en Red de Cáncer (CIBERONC); Columbia University; Health and Medical University; German Cancer Research Center (DKFZ); Faculty of Medicine and University Hospital Cologne, University of Cologne; University of Southern California; German Cancer Research Center (DKFZ); University of Cambridge; University of Eastern Finland; Dr. Margarete Fischer-Bosch-Institute of Clinical Pharmacology; The University of Melbourne; The Institute of Cancer Research; University of Manchester; University of Sydney; Peter MacCallum Cancer Center; Pomeranian Medical University; University Hospital Ulm; Lund University; Stanford University School of Medicine; The Institute of Cancer Research; The Institute of Cancer Research; Oslo University Hospital and University of Oslo; Stanford Cancer Institute, Stanford University School of Medicine; KU Leuven; University of Hawaii Cancer Center; Karolinska Institutet; Pomeranian Medical University; University Hospital Erlangen; University of Eastern Finland; University Hospital of Heraklion; University of Toronto; University of Helsinki; University of Helsinki; Leuven Cancer Institute, University Hospitals Leuven; Leuven Cancer Institute, University Hospitals Leuven; University of Manchester, Manchester Academic Health Science Centre; University Medical Center Hamburg-Eppendorf; Memorial Sloan Kettering Cancer Center; University of North Carolina at Chapel Hill; Hannover Medical School; American Cancer Society; IFOM ETS - The AIRC Institute of Molecular Oncology; Peter MacCallum Cancer Centre; MASA; Mayo Clinic; University of Westminster; University of Oulu; University Hospital Ulm; Fondazione IRCCS Istituto Nazionale dei Tumori (INT); German Cancer Research Center (DKFZ); University of Cambridge; Memorial Sloan Kettering Cancer Center; Hospital Universitario Puerta de Hierro; University Hospital of Larissa; King’s College London; Faculty of Medicine and University Hospital Cologne, University of Cologne; University Hospital Ulm; Mayo Clinic; University of Cambridge; Ss. Cyril and Methodius University in Skopje, Medical Faculty, University Clinic of Radiotherapy and Oncology; Monash University; University of Southampton; American Cancer Society; Leiden University Medical Center; The Institute of Cancer Research; The University of Edinburgh; University of North Carolina at Chapel Hill; Mayo Clinic; University of Manchester, Manchester Academic Health Science Centre; University of Cambridge; Karolinska Institutet; Leuven Cancer Institute, University Hospitals Leuven; University of Oulu; University of California Irvine; Karolinska Institutet; University of Cambridge; The Netherlands Cancer Institute - Antoni van Leeuwenhoek hospital; Erasmus MC Cancer Institute; The Netherlands Cancer Institute - Antoni van Leeuwenhoek hospital; Erasmus MC Cancer Institute

**Keywords:** CHEK2 c.1100delC germline genetic variant, radiotherapy, systemic treatment, contralateral breast cancer risk, survival

## Abstract

Breast cancer (BC) patients with a germline *CHEK2* c.1100delC variant have an increased risk of contralateral BC (CBC) and worse BC-specific survival (BCSS) compared to non-carriers. We aimed to assess the associations of *CHEK2* c.1100delC, radiotherapy, and systemic treatment with CBC risk and BCSS.

Analyses were based on 82,701 women diagnosed with invasive BC including 963 *CHEK2* c.1100delC carriers; median follow-up was 9.1 years. Differential associations of treatment by *CHEK2* c.1100delC status were tested by including interaction terms in a multivariable Cox regression model. A multi-state model was used for further insight into the relation between *CHEK2* c.1100delC status, treatment, CBC risk and death.

There was no evidence for differential associations of therapy with CBC risk by *CHEK2* c.1100delC status The strongest association with reduced CBC risk was observed for the combination of chemotherapy and endocrine therapy [HR(95%CI): 0.66 (0.55–0.78)]. No association was observed with radiotherapy. Results from the multi-state model showed shorter BCSS for *CHEK2* c.1100delC carriers versus non-carriers also after accounting for CBC occurrence [HR(95%CI) :1.30 (1.09–1.56)].

In conclusion, systemic therapy was associated with reduced CBC risk irrespective of *CHEK2* c.1100delC status. Moreover, *CHEK2* c.1100delC carriers had shorter BCSS, which appears not to be fully explained by their CBC risk.

## Introduction

Breast cancer (BC) has the highest incidence in women worldwide^1^. One of the germline variants that confer a moderate increased BC risk is the *CHEK2* c.1100delC variant^2–4^, which is found in approximately 0.7% of the Northern and Western European populations^5^. Overall, carriers of this variant are diagnosed at a younger age than non-carriers^4^ and the majority develops BCs that are estrogen receptor (ER)- and progesterone receptor (PR)-positive and human epidermal growth factor receptor 2 (HER2)-negative^3, 6^. Although this BC subtype has the most favorable prognosis in the general BC population^7^, *CHEK2* c.1100delC carriers have a higher risk of developing contralateral breast cancer (CBC) and worse survival^3, 4, 6, 8, 9^ compared to non-carriers.

Reasons behind these differences are still unclear. A possible explanation is that *CHEK2* c.1100delC carriers have a different response to treatment compared to non-carriers, e.g., their normal tissue might experience more harm from radiotherapy. *CHEK2* c.1100delC carriers have a functional deficiency in checkpoint kinase 2 (CHK2), a kinase that controls phosphorylation of downstream factors, such as BRCA1 and BRCA2^10^. This leads to a reduced BRCA1/2 function, impaired DNA repair and increased risk of BC^11^. A recent study showed that the *CHEK2* c.1100delC variant also disrupts the apoptosis of BC cells, causing unchecked proliferation and contributing to a poorer prognosis^12^. Radiotherapy has been shown to increase the risk of CBC in the general BC population, especially in younger patients^13^. Treatment with radiotherapy causes DNA strand breaks, which are less likely to be repaired in *CHEK2* c.1100delC carriers^14^. While this might be beneficial for the treatment of the first primary cancer, which is likely to have lost both functional *CHEK2* alleles, and cannot repair DNA strand breaks at all, carriers might be more prone to developing a CBC^15^. One case-only study showed a non-significant increased risk for developing CBC after treatment with radiotherapy in *CHEK2* c.1100delC carriers versus non-carriers but due to the small study size the effects in the younger population could not be investigated^16^. Only one other small study reported on the association between radiotherapy and CBC risk by *CHEK2* c.1100delC status^8^.

On the other hand, less is known about whether the effects of systemic therapy on CBC risk and survival differ by *CHEK2* c.1100delC status. A population-based study showed a significant decrease in CBC risk following chemotherapy and endocrine therapy in general BC^17^. One single-hospital study also found a decreased risk of CBC after chemotherapy use in *CHEK2* c.1100delC carriers, and did not find evidence for a differential association by *CHEK2* c.1100delC status^18^. This study also found no evidence for a differential impact of chemotherapy on survival^18^.

Given this uncertainty, our aim was to assess, within a large international cohort, potential differential associations of treatment given for the first primary BC (i.e. radiotherapy, chemotherapy and endocrine therapy) by *CHEK2* c.1100delC status with CBC risk, and to investigate whether the worse breast cancer-specific survival (BCSS) so far reported in carriers is explained solely by the increased CBC risk.

## Materials And Methods

### Study population

**Figure 1:**
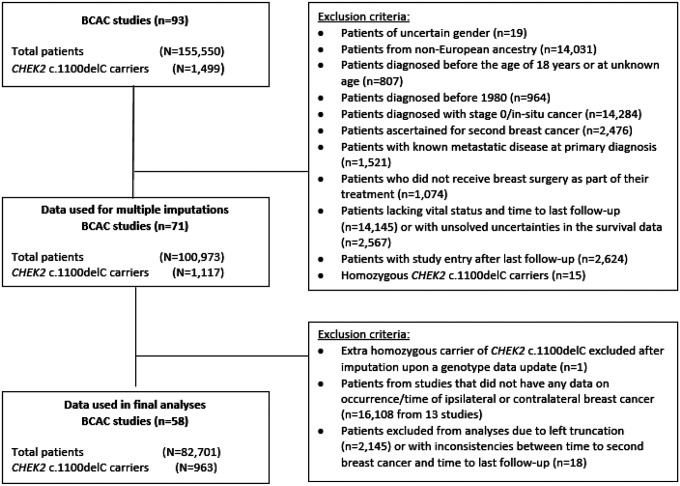
Data flowchart of inclusion and exclusion of patients with breast cancer from the Breast Cancer Association Consortium (BCAC) database

We used data from the Breast Cancer Association Consortium (BCAC), selected women of European ancestry, diagnosed with a first primary invasive BC between 1980 and 2018; exclusion criteria are shown in Fig. 1. The main analyses were based on 82,701 BC patients from 58 BCAC studies (Table S1). All individual studies were approved by the appropriate institutional review boards and/or medical ethical committees. Written informed consent was obtained from all study participants.

Previous analyses investigating the relationship between *CHEK2* c.1100delC status, risk of CBC, and mortality have been based on a subset of patients genotyped with Taqman^3, 4^. In particular, the current study includes most carriers from the Weischer et al. study (n = 459)^4^ and from the Kriege et al. study (n = 193)^18^, but is based on a larger number of BC patients and includes updated follow-up data.

### Data collection

Data included information about *CHEK2* c.1100delC status, vital status at last follow-up, CBC occurrence, age and year of diagnosis of the first primary BC, tumor characteristics of the first primary BC and CBC, as well as treatment given for the first primary BC (Tables 1 and 2). In particular, all relevant clinical-pathological and treatment information, as well as outcome information, was collected by individual studies and harmonized by the BCAC Survival, Pathology and Treatment Working Group at the Netherlands Cancer Institute, Amsterdam, the Netherlands, in collaboration with the individual studies before incorporation into the BCAC database (version 13, May 2021). *CHEK2* c.1100delC status was obtained from five different sources: BRIDGES sequencing data^19^, Taqman and iPLEX genotyping^3, 4, 20^, and imputed genotypes from OncoArray^21^ or iCOGS^22^ as described in the Supplementary Methods.

### Statistical analyses

Multiple imputation, performed using R package MICE (version 3.13.0), was used to handle missing values in clinical and pathological variables. Details are given in the Supplementary Methods and Table S2. Descriptive statistics are shown as mean ± standard deviation (SD) or median and interquartile range (IQR). We used Pearson’s χ^2^ test for categorical data and Kruskal-Wallis test for continuous data to calculate differences in patients’ characteristics. The primary study outcomes were time to CBC and BCSS (time to death due to BC).

Hazard ratios (HRs) and 95% confidence intervals (CIs) for the association of treatment given for the first primary BC (radiotherapy and/or type of systemic treatment) and *CHEK2* c.1100delC status with time to CBC were estimated via Cox regression models allowing for delayed entry, stratified by country and adjusted for age at first primary BC diagnosis, tumor size, nodal status, grade and ER status. Since ER status is known to violate the proportionality hazards assumption and because the majority of *CHEK2* c.1100delC carriers develop ER-positive BC, we performed an additional main analysis restricted to patients diagnosed with a first primary ER-positive BC. We assumed that patients with unknown CBC status did not develop a CBC during follow-up, and that for CBC cases with unknown time from first primary BC to CBC diagnosis, CBC occurrence was at last available follow-up.

Time at risk started either three months after first primary BC diagnosis or at study entry if entry was more than three months after first primary BC diagnosis, and ended at time of CBC, death or last follow-up, whichever came first. We tested for potential differential association of adjuvant and/or neo-adjuvant therapy on CBC risk according to *CHEK2* c.1100delC status by including an interaction term between treatment (radiotherapy or systemic treatment) variable and *CHEK2* c.1100delC status in the model. CBC risk analyses were stratified by two follow-up time intervals: i) the first 5 years after BC diagnosis and ii) starting 5 years after BC diagnosis.

**Figure 2:**
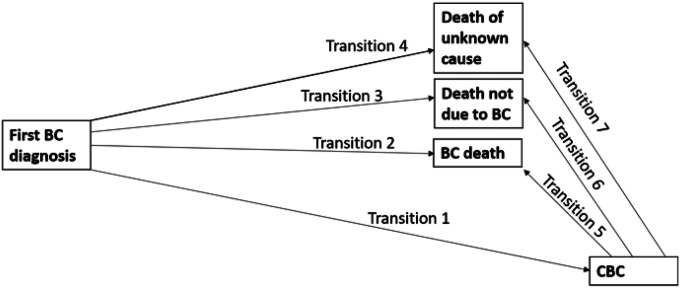
Graphical representation of the multi-state model Abbreviations: BC=breast cancer, CBC=contralateral breast cancer

To gain further insight into the relation between *CHEK2* c.1100delC status, treatment given for the first primary BC, CBC risk and death, we used a multi-state model in the framework of the Cox model, with diagnosis of the first primary BC as initial state, diagnosis of CBC as intermediate (transient) state, and death due to BC, death due to other causes, and death due to unknown causes as absorbing states (Fig. 2), as specified in the Supplementary Methods.

The main CBC risk and multi-state analyses were performed on imputed datasets. Complete-case analyses (excluding study subjects with missing values in any of the variables included in the models) were performed as sensitivity analyses. Additional analyses were restricted to: a) patients diagnosed with first primary BC from 2000 onwards to reduce heterogeneity in treatment regimens; b) patients diagnosed at age 40 or younger to see if the association with radiotherapy was stronger in this subgroup, as reported previously in the general BC population^13^.

## Results

Patients carrying the *CHEK2* c.1100delC variant were diagnosed at a younger age and earlier years. The tumors of carriers were larger at time of diagnosis, were more often lymph node-positive, grade 2, and ER- and PR-positive than in non-carriers; also some differences in treatment were observed (Table 1).

### Contralateral breast cancer

*CHEK2* c.1100delC carriers were diagnosed at younger age and in earlier calendar years, both for the first primary tumor as well as for the CBC. Overall, the characteristics of the CBC were similar between the non-carriers and carriers (Table S3). However, *CHEK2* c.1100delC carriers more often had positive nodes at CBC diagnosis than non-carriers (p = 0.02).

### CBC risk by treatment and *CHEK2* c.1100delC carrier status

There was no evidence for a differential association of *CHEK2* c.1100delC status by radiotherapy [Tables 2–3: P-value for interaction = 0.31 in all patients and P-value for interaction = 0.99 in ER-positive patients] or systemic therapy [P-value for interaction = 0.46 in all patients and P-value for interaction = 0.68 in ER-positive patients]. Moreover, we did not find an association with radiotherapy on CBC risk [HR (95%CI): 1.07 (0.94–1.21), P = 0.33 in all BC patients and 1.07 (0.92–1.25), P = 0.35 in ER-positive BC patients]. Regarding systemic therapy, we observed that chemotherapy alone [HR (95%CI): 0.77 (0.62–0.96), P = 0.02 in all BC patients and 0.73 (0.52–1.03), P = 0.07 in ER-positive BC patients], endocrine therapy alone [HR (95%CI): 0.70 (0.58–0.83), P < 0.001 in all BC patients and 0.66 (0.54–0.81), P < 0.001 in ER-positive BC patients] and the combination of both [HR (95%CI): 0.65 (0.55–0.78), P < 0.001 in all BC patients and 0.65 (0.52–0.82), P < 0.001 in ER-positive BC patients] had a protective association with CBC risk compared to women who did not receive any systemic therapy as part of their treatment.

Results of analyses for patients diagnosed at the age of 40 years or younger or for patients diagnosed from 2000 onwards were in line with the results of the main analyses (Tables S4-S5). Complete-case analyses results were consistent with the corresponding results of the imputed data analyses (Tables S6-S9), except for the association with radiotherapy in patients diagnosed at the age of 40 years or younger. For these patients, radiotherapy was significantly associated with increased CBC risk in the complete-case analysis with follow-up starting 5 years after diagnosis of the first primary BC [Table S7; HR (95%CI): 2.12 (1.06–4.22), P = 0.03]. In addition, interaction terms between treatments and *CHEK2* c.1100delC status could not be properly estimated in some of the complete-case analyses, due to insufficient data. These included, among others, the analysis based on all patients with follow-up starting at 5 years after BC diagnosis; the analysis restricted to patients diagnosed at the age of 40 years or younger and based on the total follow-up; and the analysis restricted to ER-positive BC with follow-up starting 5 years after BC diagnosis (Tables S10-S12).

### CHEK2 c.1100delC carrier status, CBC and survival trajectories

*CHEK2* c.1100delC carriers versus non-carriers had an almost 2.4 fold risk of developing a CBC [HR (95%CI): 2.37 (1.82–3.08), P < 0.001 in all patients and 2.55 (1.87–3.48), P < 0.001 in patients with an ER-positive first primary BC; Table 4] and a 1.3-fold risk of BC death after censoring for CBC occurrence [HR (95%CI): 1.30 (1.09–1.56), P = 0.003 in all patients and 1.38 (1.12–1.71), P = 0.003 in patients with an ER-positive first primary BC; Table 4]. There was no evidence for association of *CHEK2* c.1100delC carrier status with other transitions. Results from the analyses restricted to patients diagnosed with first primary BC from 2000 onwards were in line with the results from the main analyses (Table S15).

Regarding treatment, radiotherapy was associated with a protective association against death due to causes other than BC or unknown causes, while there was no significant association with BC-specific death (Tables S13-S15). Endocrine therapy alone was associated with a significantly decreased risk of BC-specific death (particularly in patients diagnosed with an ER-positive first primary BC) and with a highly significantly decreased risk of death due to unknown causes. The combination of endocrine therapy and chemotherapy was associated with decreased risk of BC death (in patients diagnosed with an ER-positive first primary BC), with risk of death due to causes other than BC and had the strongest protective association against death due to unknown causes (Tables S14). The corresponding complete-case analyses showed similar patterns of association (Tables S16-S18).

## Discussion

The main goal of this study was to assess potential differential associations of treatment by *CHEK2* c.1100delC status with CBC risk, and to investigate if the poorer survival in *CHEK2* c.1100delC carriers may be explained alone by the occurrence of CBC. The Breast Cancer Association Consortium provided a unique resource of 963 carriers of this single *CHEK2* variant to study this question in more detail.

These data did not support the hypothesis of differential associations of treatment with CBC risk by *CHEK2* c.1100delC status. As expected, systemic therapy was found to decrease CBC risk, with the strongest association in the first five years after first primary BC diagnosis, when endocrine therapy is likely to be ongoing^17, 23^. Overall, we did find that the combination of endocrine therapy with chemotherapy resulted in the largest reduction in CBC risk, which has been previously reported^17^. The lack of evidence for a differential association of systemic therapy with CBC risk by *CHEK2* c.1100delC status suggests that carriers experience a similar beneficial effect as non-carriers. This is in line with previous studies in *CHEK2* c.1100delC carriers^18, 24, 25^.

Also, we did not find a significant association of radiotherapy with CBC risk. This lack of association is in contrast with previous studies in sporadic BC patients, which showed that radiotherapy is a contributor to CBC risk, especially when treatment was administered at a younger age^13, 26–28^. One explanation for this might be the change of radiation techniques over time. However, analyses restricted to patients diagnosed from the year 2000 onwards, when treatment regimens were expected to be more homogeneous, showed similar results as were found in the main analyses. Therefore, although observational – and non-randomized – studies like the present cannot rebut hypotheses of causality, these changes are unlikely to be the reason behind the lack of association between radiotherapy and CBC risk in our study.

In line with previous studies^3, 4^ we found a greater than two-fold increased risk of CBC in *CHEK2* c.1100delC carriers compared to non-carriers. This is consistent with the reported increase in risk of a first primary BC^2, 19^, suggesting that genetic variants that predispose to the development of a first primary BC will also predispose to the development of a CBC. We also observed a shorter BCSS in *CHEK2* c.1100delC carriers compared to non-carriers, after accounting for CBC occurrence, age at diagnosis of the first primary BC and tumor characteristics. This suggests that the shorter BCSS in *CHEK2* c.1100delC carriers versus non-carriers is partly explained by a component other than the established prognostic factors. Moreover, *CHEK2* c.1100delC carriers were on average diagnosed in earlier calendar years compared to non-carriers. Therefore, carriers probably received less efficacious chemotherapy and endocrine therapy compared to non-carriers, which could have affected survival.

The main strengths of our study are the large sample size, including information about tumor pathology, treatment, time to CBC and survival, and a median follow-up of over 9 years. In addition, the use of a multi-state model provides important advantages compared to individual survival models with different endpoints. By modeling all events of interest together, the multi-state model gives insight on how intermediate events, such as CBC, affects survival. Moreover, it allows estimation of transition-specific treatment and covariates effects, thereby providing insight on whether and to what extent the effects change across transitions and corresponding endpoints. Most of the studies were hospital or population based and most BC patients unaware of a *CHEK2* variant, which we determined in the research setting. Therefore, it is highly unlikely that knowledge of carrier status could have affected clinical data collection.

There are some limitations to our study that need to be acknowledged. Between studies there was minor heterogeneity in the definition of stage, grade and cut-offs for ER, PR, and HER2 status, which would have affected both carriers and non-carriers to a similar extent and is unlikely to have impacted our conclusions. Many of the variables related to tumor characteristics and treatment had large proportions of missing values. Complete-case analyses have less power to detect the associations of interest and might be biased if case data are not missing completely at random^29^. We addressed the missing data problem by employing multiple imputation^29^, which should provide unbiased estimates, provided data are missing at random and that imputation models are correctly specified. Analyses restricted to complete-case data yielded results that were mostly consistent with the results based on imputed data. In addition, in some complete-case analyses the number of *CHEK2* c.1100delC carriers was too low to properly estimate the interaction terms. This underlines the importance of the analyses based on imputed data, which avoids losses in the number of cases and events in the analyses. We also did not consider type of chemotherapy or endocrine therapy in the analyses, nor had we information about ovarian function suppression. Moreover, information about the occurrence of primary ipsilateral BCs was very limited and could not be properly accounted for in our analyses. However, based on the available information, there was no difference in the proportion of ipsilateral BC between *CHEK2* c.1100delC carriers and non-carriers (0.6% in both groups) and is unlikely to have had a major impact on our BCSS results. An additional limitation was the lack of information on cause of death for about 25% of those who had died. This would result in a loss of power to detect associations with BCSS in case most of the deaths of unknown causes were due to BC. However, this would, at worst, dilute our results rather than leading to false-positive significant associations with BCSS. Finally, while we accounted for several established BC prognostic factors in our analyses, we cannot exclude the presence of residual bias affecting to some extent our results. An example of such bias is known as “indication bias”, which applies to the presence of an indication which causes or affects the outcome of interest^30^. This could explain some of the unexpected results for the association of radiotherapy and systemic treatment with death-related outcomes, in case treatment decisions are influenced by the presence/absence of certain conditions or morbidities in such a way that patients receiving the treatment are less likely to die from other causes than BC. While indication bias could have affected the treatment-related effects on mortality, it is less likely to be an issue for the association of *CHEK2* c.1100delC status and treatment with CBC risk and survival.

In conclusion, the results of our study did not provide evidence for differential associations with radiation or systemic therapy by *CHEK2* c.100delC status on CBC risk. This suggests that associations with these treatments on CBC risk are similar between carriers and non-carriers. Furthermore, we confirmed the presence of a risk component for BC-specific death in *CHEK2* c.1100delC carriers which is not explained by CBC occurrence or characteristics of the first primary BC. Genotyping of *CHEK2* c.1100delC in patients of ongoing clinical trials would allow the evaluation of treatment response in detail and determine any impact of the *CHEK2* c.1100delC variant on the efficacy of BC treatment. In addition, studies focusing on for examples the molecular copy number aberration pro le of *CHEK2*-related tumors should further shed light on potential biological mechanisms underlying the observed increased CBC risk and possible worse survival in *CHEK2* c.1100delCcarriers.

## Figures and Tables

**Table 1:** Clinical, tumor and treatment characteristics for the first primary BC by *CHEK2* c.1100delC carrier status.

Characteristics	Non-carriers	*CHEK2* c.1100delC carriers	P-value
Number of patients, n	81,738	963	
Number of patients diagnosed with CBC, n	1,757 (2.1)	59 (6.1)	
Number of patients diagnosed with ipsilateral BC, n (%)*	517 (0.6)	6 (0.6)	
Total FU time, years (IQR)	9.2 (5.3–13.6)	9.6 (5.5–13.9)	
Clinical risk factors			
Age at diagnosis, y, median (IQR)	56 (47–64)	52 (44–61)	<0.001
Age at diagnosis, n (%)			<0.001
< 40 years	9,471 (11.6)	171 (17.8)	
40–50 years	19,978 (24.4)	277 (28.8)	
50–60 years	23,044 (28.2)	266 (27.6)	
> 60 years	29,245 (35.8)	249 (25.9)	
Year of diagnosis, n (%)			<0.001
1980–1989	2,259 (2.8)	48 (5.1)	
1990–1999	20,055 (24.8)	297 (31.3)	
2000–2009	45,910 (56.7)	492 (51.8)	
≥ 2010	12,781 (15.8)	113 (11.9)	
Missing, n	733	13	
Tumor characteristics			
Tumor size, n (%)			0.01
≤ 2 cm	40,263 (63.0)	421 (58.6)	
> 2 and ≤ 5 cm	20,977 (32.8)	273 (38.0)	
> 5 cm	2,718 (4.3)	24 (3.3)	
Missing, n	17,780	245	
Lymph node status, n (%)			<0.001
Negative	42,079 (61.4)	439 (54.8)	
Positive	26,456 (38.6)	362 (45.2)	
Missing, n	13,203	162	
Grade, n (%)			0.01
Grade 1	12,572 (19.1)	112 (15.3)	
Grade 2	31,594 (48.1)	388 (53.0)	
Grade 3	21,536 (32.8)	232 (31.7)	
Missing, n	16,036	231	
Morphology, n (%)			0.16
Ductal	52,127 (74.0)	659 (77.5)	
Lobular	10,596 (15.0)	116 (13.7)	
Medullary	619 (0.9)	3 (0.4)	
Mixed (ductal & lobular)	3,032 (4.3)	37 (4.4)	
Mucinous	895 (1.3)	7 (0.8)	
Papillary	160 (0.2)	22 (0.1)	
Tubular	908 (1.3)	1 (0.6)	
Other	2,111 (3.0)	5 (2.6)	
Missing, n	11,290	113	
ER status, n (%)			<0.001
Negative	13,918 (20.4)	93 (11.8)	
Positive	54,481 (79.7)	694 (88.2)	
Missing, n	13,339	176	
PR status, n (%)			<0.001
Negative	19,128 (32.1)	169 (24.5)	
Positive	40,548 (68.0)	520 (75.5)	
Missing, n	22,062	274	
HER2 status, n (%)			0.55
Negative	37,395 (83.5)	418 (82.5)	
Positive	7,376 (16.5)	89 (17.6)	
Missing, n	36,967	456	
Treatment			
Surgery, n (%)			<0.001
Breast conserving surgery	23,706 (43.3)	244 (36.3)	
Mastectomy	16,129 (29.4)	259 (38.5)	
Type unknown	15,330 (27.6)	169 (25.2)	
Missing, n	26,573	291	
Radiotherapy, n (%)			0.36
No	13,163 (26.0)	181 (27.6)	
Yes	37,479 (74.0)	474 (72.4)	
Missing, n	31,096	308	
Systemic therapy, n (%)			<0.001
No systemic therapy	4,996 (11.2)	94 (17.0)	
CT, no ET	7,501 (16.8)	88 (15.9)	
ET, no CT	16,976 (38.1)	153 (27.7)	
Both CT and ET	15,116 (33.9)	218 (39.4)	
Missing, n	37,149	410	
Trastuzumab, n (%)			0.96
No	37,466 (95.4)	478 (95.2)	
Yes	1,819 (4.6)	24 (4.8)	
Missing, n	42,453	461	

Percentages are only on observed, non-missing data, and may not total 100 because of rounding.

* Data component not actively collected in BCAC. Abbreviations: CBC = contralateral breast cancer; CT = chemotherapy; ER = estrogen receptor; ET = endocrine therapy; PR = progesterone receptor; HER2 = human epidermal growth factor receptor 2.

**Table 2:** Contralateral breast cancer risk (hazard ratio) by treatment for first primary breast cancer and *CHEK2* c.1100delC status. Stratified by time since first primary breast cancer diagnosis.

	Total follow-up time			< 5-year follow-up			> 5 years follow-up		
No of patients	82,701			73,354			62,688		
No of CBC events	1,816			656			1,160		
	HR (95%CI)	P-value	P-int	HR (95%CI)	P-value	P-int	HR (95%CI)	P-value	P-int
*CHEK2* c.1100delC status	2.37 (1.82–3.08)	<0.001		3.08 (2.12–4.48)	<0.001		1.93 (1.33–2.80)	<0.001	
Radiotherapy			0.31			0.30			0.77
No radiotherapy	ref			ref			ref		
Radiotherapy	1.07 (0.94–1.21)	0.33		0.98 (0.81–1.19)	0.84		1.12 (0.96–1.31)	0.16	
Systemic therapy			0.46			0.70			0.39
No systemic therapy	ref			ref			ref		
CT, no ET	0.77 (0.62–0.96)	0.02		0.58 (0.41–0.83)	0.003		0.90 (0.70–1.15)	0.39	
ET, no CT	0.70 (0.58–0.83)	<0.001		0.62 (0.46–0.84)	0.002		0.73 (0.59–0.91)	0.005	
Both CT and ET	0.65 (0.55–0.78)	<0.001		0.50 (0.37–0.68)	<0.001		0.75 (0.62–0.93)	0.007	

Adjusted for age at diagnosis, ER status, tumor size, nodal status and grade of first primary breast cancer. Abbreviations: CT = chemotherapy; ET = endocrine therapy; P-int = P-value for the comparison of a model including an interaction term between *CHEK2* c.1100delC status and a specific treatment (radiation or systemic treatment) with a model without any interaction term.

**Table 3:** Contralateral breast cancer risk (hazard ratio) by treatment for first primary BC and *CHEK2* c.1100delC status in ER-positive BC patients. Stratified by time since first primary breast cancer diagnosis.

	Total follow-up time			< 5-year follow-up			> 5 years follow-up		
No of patients	55,175			51,146			41,269		
No of CBC events	1,133			427			706		
	HR (95%CI)	P-value	P-int	HR (9 5% Cl)	P-value	p-int	HR (95%CI)	P-value	P-int
*CHEK2* c.1100delC status	2.55 (1.87–3.48)	<0.001		3.42 (2.24–5.22)	<0.001		1.94 (1.22–3.08)	0.005	
Radiotherapy			0.99			0.47			0.41
No radiotherapy	ref			ref			ref		
Radiotherapy	1.07 (0.92–1.25)	0.35		1.04 (0.81–1.34)	0.75		1.09 (0.90–1.32)	0.36	
Systemic therapy			0.68			0.91			0.96
No systemic therapy	ref			ref			ref		
CT, no ET	0.73 (0.52–1.03)	0.07		0.62 (0.38–1.03)	0.06		0.80 (0.52–1.23)	0.31	
ET, no CT	0.66 (0.54–0.81)	<0.001		0.55 (0.40–0.77)	<0.001		0.73 (0.57–0.94)	0.02	
Both CT and ET	0.65 (0.52–0.82)	<0.001		0.48 (0.34–0.69)	<0.001		0.77 (0.58–1.03)	0.08	

Adjusted for age at diagnosis, nodal status, size category and grade of first primary breast cancer. Abbreviations: CT = chemotherapy; ET = endocrine therapy; P-int = P-value for the comparison of a model including an interaction term between *CHEK2* c.1100delC status and a specific treatment (radiotherapy or systemic treatment) with a model without any interaction term.

**Table 4:** Multi-state model in all breast cancer patients and in patients diagnosed with a first primary ER-positive breast cancer: Hazard ratio for the comparison of *CHEK2* c.1100delC carriers versus non-carriers for each transition.

Analysis	Transition	Description	HR (95% CI)	P	Cases	Events
All BC patients	1	First primary BC -> CBC	2.37 (1.82–3.08)	<0.001	82,701	1,816
	2	First primary BC -> BC death	1.30 (1.09–1.56)	0.003		7,467
	3	First primary BC -> death not due to BC	1.00 (0.75–1.34)	0.98		4,247
	4	First primary BC -> death of unknown cause	1.07 (0.76–1.49)	0.70		3,548
	5	CBC -> BC death	1.23 (0.72–2.10)	0.46	1,816	281
	6	CBC -> death not due to BC	0.60 (0.14–2.52)	0.49		124
	7	CBC -> death of unknown cause	1.21 (0.41–3.53)	0.73		94
Patients diagnosed with primary ER-positive BC	1	First primary BC -> CBC	2.55 (1.87–3.48)	<0.001	55,175	1,133
	2	First primary BC -> BC death	1.38 (1.12–1.71)	0.003		4,266
	3	First primary BC -> death not due to BC	1.13 (0.81–1.56)	0.47		2,817
	4	First primary BC -> death of unknown cause	0.97 (0.63–1.48)	0.87		2,090
	5	CBC -> BC death	1.49 (0.79–2.81)	0.21	1,133	167
	6	CBC -> death not due to BC	0.89 (0.20–4.06)	0.89		80
	7	CBC -> death of unknown cause	0.61 (0.14–2.79)	0.53		55

Abbreviations: HR = hazard ratio; CI = confidence interval; BC = breast cancer; CBC = contralateral breast cancer. The models included age at first primary BC diagnosis, nodal status, tumor size, grade, radiotherapy and systemic treatment given for the first primary BC as covariates. The model based on all BC patient included ER status of the first primary BC as additional covariate. Baseline hazards were allowed to vary across country and transition. All the estimates from the model are shown in Tables S13-S14.

## Data Availability

The datasets analyzed during the current study are not publicly available due to protection of participant privacy and confidentiality, and ownership of the contributing institutions, but may be made available in an anonymized form via the corresponding author on reasonable request and after approval of the involved institutions. To receive access to the data, a concept form must be submitted, which will then be reviewed by the BCAC Data Access Coordination Committee (DACC); see http://bcac.ccge.medschl.cam.ac.uk/bcacdata/.

## Novelty And Impact

Based on 82,701 breast cancer patients, including 963 *CHEK2* c.1100delC carriers we found no differential association of radiotherapy or systemic treatment with contralateral breast cancer risk by *CHEK2* c.1100delC status. We observed a shorter breast cancer-specific survival for *CHEK2* c.1100delC carriers, after accounting for contralateral breast cancer occurrence and characteristics of the first breast cancer. This work may inform decisions concerning the harms and benefits of treatment of *CHEK2* c.1100delC carriers in clinical practice.

## Abbreviations

BC: breast cancer
BCAC: Breast Cancer Association Consortium
BCSS: breast cancer-specific survival
CBC: contralateral breast cancer
CI: confidence interval
CMF: Cyclophosphamide Methotrexate Fluorouracil
CT: chemotherapy
ER: estrogen receptor
ET: endocrine therapy
HER2: human epidermal growth factor receptor 2
HR: hazard ratio
IQR: interquartile range
P: p-value
PR: progesterone receptor
SD: standard deviation
